# Physical Properties of Bacterial Nanocellulose as an Encapsulant Material of Vitamin B12

**DOI:** 10.3390/molecules30214172

**Published:** 2025-10-23

**Authors:** Hasbleidy Palacios-Hinestroza, María Camila López-Jaramillo, Julián Paul Martínez-Galán, Carlos Molina-Ramírez, Diego Mauricio Sánchez-Osorno

**Affiliations:** 1 Department of Basic Sciences, Campus Tlajomulco, University of Guadalajara, Tlajomulco de Zúñiga 45641, Mexico; hasbleidy.palacios@academicos.udg.mx; 2 Grupo de Investigación e Innovación Ambiental (GIIAM), Institución Universitaria Pascual Bravo, Cl. 73, No. 73a-226, Medellín 050034, Colombia; m.lopezja@pascualbravo.edu.co; 3 Grupo de Investigación Alimentación y Nutrición Humana (GIANH), Escuela de Nutrición y Dietética, Universidad de Antioquia, Cl. 67, No. 53-108, Medellín 050010, Colombia; julian.martinez@udea.edu.co; 4 Grupo de Investigación en Química y Bioprospección de Productos Naturales (QUIBIP), Universidad del Magdalena, Cl. 29H3, No. 22-01, Santa Marta 470004, Colombia; cmolinar@unimagdalena.edu.co; 5 Grupo de Investigación en Calidad y Productividad (QUALIPRO), Institución Universitaria Pascual Bravo, Cl. 73, No. 73a-226, Medellín 050034, Colombia

**Keywords:** vitamin B12, encapsulation, bacterial nanocellulose, maltodextrin, spray drying, powder flowability, thermal stability, ATR-FTIR, rheology, microstructure

## Abstract

This study presents a comprehensive comparison of bacterial nanocellulose (BNC) and maltodextrin (MDX) as encapsulating agents for vitamin B12, using spray drying. The research focuses on the physical powder characteristics, such as flowability and cohesion, which are critical for industrial applications. The encapsulation of vitamin B12 was confirmed by ATR-FTIR analysis, which showed characteristic band shifts at 2138 cm^−1^ indicating interaction between the vitamin and the encapsulant matrices. Powder flow analysis revealed that BNC-based powders exhibited lower cohesion (CI = 13.3) and better flowability compared to MDX-based powders (CI = 7.7–13.7). Scanning electron microscopy (SEM) showed that all powders consisted of micrometric spherical particles ranging from 2 to 8 µm, with BNC producing particles with a more defined and less agglomerated structure. Thermogravimetric analysis (TGA) demonstrated that both matrices enhanced the thermal stability of vitamin B12, with BNC increasing the degradation onset temperature from 200 °C to 260 °C and the maximum degradation temperature from 274 °C to 317 °C providing a slightly higher onset degradation temperature. The results suggest that BNC is a promising alternative to traditional encapsulants like maltodextrin, offering up to 43 °C higher thermal protection and improved physical properties for encapsulating thermosensitive compounds in dietary applications.

## 1. Introduction

Vitamins are essential micronutrients crucial for maintaining health, and their deficiency can lead to various diseases. Vitamin B12, or cyanocobalamin, is a water-soluble vitamin vital for neurological function and the formation of red blood cells. However, its susceptibility to degradation by heat, light, and oxygen poses a significant challenge for its incorporation into food and pharmaceutical products [1,2]. Encapsulation is a widely used technique to protect sensitive compounds like vitamin B12 from degradation, thereby enhancing their stability and bioavailability [3,4].

The choice of encapsulant material is critical to the success of the encapsulation process. The ideal material should be food-grade, biodegradable, non-toxic, and capable of forming a protective barrier around the active compound. Maltodextrin (MDX), a polysaccharide derived from starch hydrolysis, is a commonly used encapsulant due to its low cost, high water solubility, and neutral taste [5]. However, its high hygroscopicity can negatively affect the stability and powder properties of the final product [6]. Powder properties, such as flowability, cohesion, and particle morphology, are critical in the food industry for efficient transport, handling, mixing, and processing operations like tableting [7,8]. For efficient operation of rapid compression equipment and for convenient handling by consumers, powders must exhibit unhindered flow [9].

Bacterial nanocellulose (BNC) has emerged as a novel and promising biopolymer for various applications, including in the food and pharmaceutical industries [7]. Produced by certain bacteria like *Komagataeibacter medellinensis*, BNC is a hydrogel characterized by its high purity and a unique supramolecular, three-dimensional network of nanosized fibers. This structure results in a large surface area, high crystallinity, a high degree of polymerization (up to 8000), and a remarkable water-holding capacity [2,8]. While some studies have explored BNC for encapsulating various compounds, including vitamins [10], a direct and detailed comparison of the powder properties of spray-dried BNC and maltodextrin for vitamin B12 encapsulation has not been reported.

This study aims to address this gap by providing the first comparative study of spray-dried BNC versus MDX for cyanocobalamin with a focus on powder flow and thermal analysis readouts. We hypothesize that BNC, owing to its unique fibrillar structure and physicochemical properties, will yield powders with superior flowability and thermal stability compared to those produced with MDX. The specific objectives are to characterize and compare the materials based on measurable outcomes, including chemical interactions (ATR-FTIR), powder flowability (Carr’s Index), particle morphology (SEM), and thermal stability (TGA).

## 2. Results and Discussion

### 2.1. ATR-FTIR

**Figure 1:**
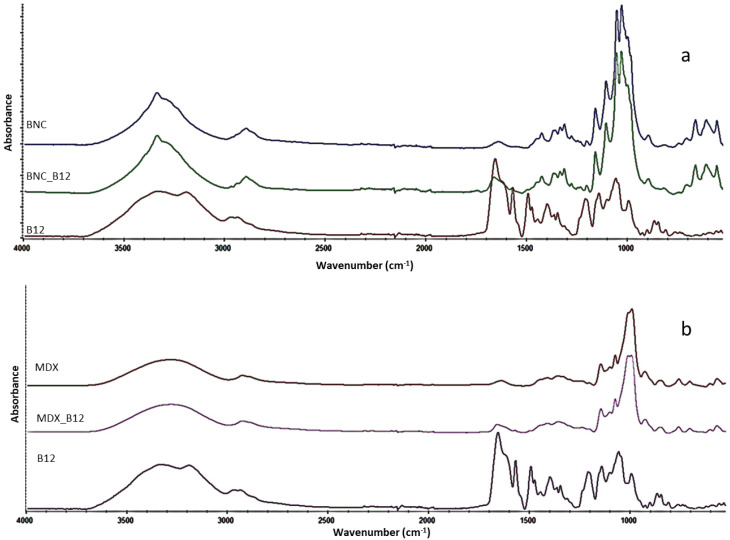
FTIR spectrum of (**a**) BNC and BNC mixed with B12 and (**b**) MDX and MDX mixed with B12.

Main bands of each pure material and the mixes with B12 are presented in Figure 1. The characteristic bands of cellulose at 3345, 2847–2921, 1162, 1110, 1059, 1035 cm^−1^ were assigned as (O-H), (C-H) in methylene and methylidyne, (C-O-C) at α-glycosidic linkage, (C-C) of the glucose ring, (C-O) of a secondary alcohol at C-2, C-3 and a primary alcohol at C-6, respectively [1]. For maltodextrin a broad band of OH stretching at 3386 cm^−1^ appeared due to hydrogen bonding of hydroxyl groups, three bands at 1023, 1080, and 1155 cm^−1^ were attributed to C-O stretching and C-O-H bending vibrations [3]. Further bands at 437, 526, 577, 608, 708, 763, 849 and 930 cm^−1^ arose from skeletal vibrations of the pyranoid ring [2,5]. The main bands of B12 are observed at 2900 cm^−1^ and 2120 cm^−1^ correspond to C–H, C≡C, respectively. Additionally, the C=O stretching vibration at 1700 cm^−1^, the C=C stretching vibration at 1580 and 1500 cm^−1^, the C–H bending vibration at 1390 cm^−1^, the C–O stretching vibration at 1220, 1140 and 1020 cm^−1^, and the characteristic C-N at 2138 cm^−1^ [6] were observed.

**Figure 2:**
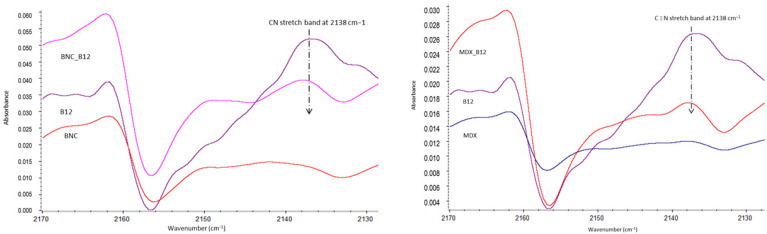
Magnification of FTIR Spectrum 2170–2130 cm^−1^.

As visible from Figure 2, the main structure of B12 is maintained when loading it onto the BNC or MDX. Characteristic bands of B12 are present in the spectra of BNC_B12 and MDX_B12. For example, the cyano group from B12 (cianocobalamin) showed no degradation of the vitamin [5]. However, a shift in the cyano band at 2138 cm^−1^ was observed, which provides evidence of the interaction of B12 with both materials, BNC and MDX.

Because of the low intensity of FTIR bandsthe B12 loading on both BNC and MDX surface does not cause a significant change in the spectra of the polymers. Therefore, in our case the network structure forming was not detectable by FTIR contrary to what was reported by Gharagozlou [5]. Main interactions between both materials and B12 are observed by shift in the peaks listed in Table 1. Interactions between BNC or MDX with B12 are possible due to hydroxyl groups present in the glucose of each material, especially the OH group at the C-6 position, which is the most accessible group for interaction, thus making the adsorption of B12 onto the material possible. Due to the strong hydrogen bonding existing in BNC, it is suggested that 40–60% of these hydroxyl groups of BNC are linked to the vitamin [6]. For B12 it is nitrogen and hydroxyl groups that are accessible for interaction with the encapsulant (BNC or MDX). Shifts in wavenumbers are observed in the mixes of both materials with B12. Those shifts in the wavenumbers correspond to OH and/or N-bearing groups. Aliphatic nitriles exhibit a typical ν(C≡N) stretching vibration around 2260–2220 cm^−1^; however, when the nitrile is aromatically conjugated or coordinated to a metal, the frequency shifts to lower wavenumbers (∼2140–2100 cm^−1^). In cyanocobalamin, the CN group is bonded to Co(III), which explains the experimental observation of the band near 2138 cm^−1^ [9]

### 2.2. Rheology of the Powders

**Figure 3:**
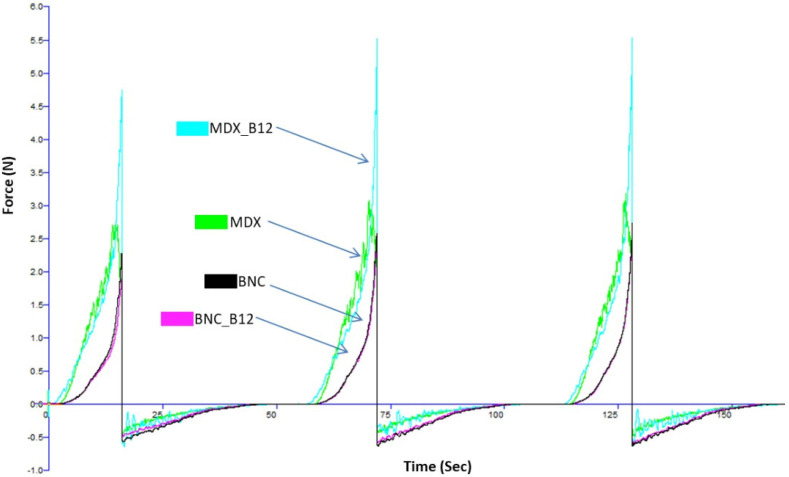
Sample flow curves for powders during three successive compressions and decompressions in the powder texturometer (MDX_B12: Cyan Color; MDX: Fluorescent Green Color; BCN: Black Color; BNC_B12: Fuchsia Color).

Powder flow refers to the movement of a collection of particles relative to one another or along the surface of the container holding them. Forces influencing this flow include gravitational force, friction, cohesion (attraction between particles), and adhesion (attraction between particles and the container walls) [26]. The sample flow curve, generated during the three-cycle compression and decompression process for each sample in the powder texturometer (without the addition of any binding liquid), is illustrated in Figure 3. The consistency in the graph patterns across the three compression and decompression cycles suggests stable powder behavior throughout the testing procedure [27]. Notably, the area representing the compression phase consistently exceeds that of the decompression phase, implying that less energy is needed for powder decompression compared to its compression [28].

**Figure 4:**
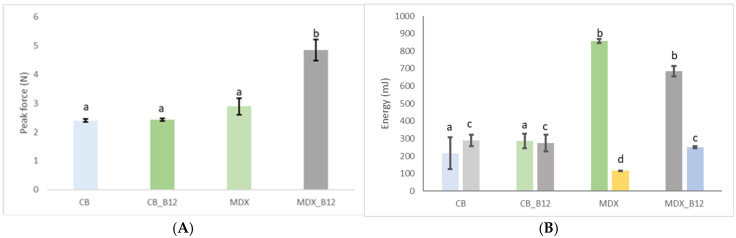
Maximum force during compression (**A**) and energy during compression ((**B**)_left) and decompression ((**B**)_right). Groups not sharing a common letter (a, b, c, d) are significantly different (*p* < 0.05).

The peak (maximum) force determined for BNC and BNC_12 is around 2.4 N. For MDX and MDX_B12 it was 2.8 N and 4.8 N, respectively. The moisture content of all powders was determined to be approximately 8% using an oven drying method at 105 °C until constant weight. It is hypothesized that the higher force peaks observed for MDX powders (Figure 4) could be related to the presence of more free water in the amorphous MDX matrix, which can act as a plasticizer and increase particle cohesion.

Additions of vitamin B12 to BNC did not increase the peak force in comparison to pure BNC powder. Conversely, it is observed that the addition of vitamin B12 to MDX increases the peak force compared with pure MDX. BNC and MDX, being hydrocolloids, readily absorb moisture. When B12 is added to the polymer system, the vitamin competes with the water molecules for the active sites of the polymer [29]. This is in accordance with the interactions observed by FTIR, showing that vitamins are absorbed by physisorption by hydrogen bonds. It observed that even when both BNC and MDX have same moisture, there is a difference between force peaks which is an indicator that free water is more abundant in MDX than BNC due to specific chemical structure of each polymer [23]. Water uptake is generally inversely proportional to molecular weight [6]. Because BNC contains a significant proportion of high-molecular-weight components, it reached moisture equilibrium faster compared to MDX, which is characterized by lower molecular weights, leading to a greater amount of unbound water [23]. Hence, MDX powders can undergo hydration that finally leads to higher peak forces, especially when mixed in MDX_B12. The high-molecular-weight fibrils of BNC rapidly achieved equilibrium, while the lower molecular weight of MDX led to higher proportions of unbound water.

This distinction is critical in encapsulation systems, since the presence of free water enhances plasticization, lowers glass transition temperature (Tg), and facilitates compaction under stress, which explains the increased resistance of MDX_B12 powders [30]. By contrast, the rigid crystalline domains of BNC, enriched in bound water, confer greater stability and limit molecular mobility, thereby explaining the minimal effect of B12 addition. This behavior aligns with reports indicating that the rheology of spray-dried hydrocolloids depends strongly on water distribution and polymer morphology rather than just total water content [31].

For BNC powders the energy for compression observed (Figure 4) shows a similar trend between both BNC and MDX powders as the trend for the peak force (F = 43.3; *p*-value = 0.000027226). Energy required for compressing the MDX powder is lower than that required for MDX_B12 but still higher that energy required for compressing BNC powders. During compression free water becomes mobile allowing the easy passage for the rotating blades during the test, which is consistent with Ghosal et al., 2010 [6].

Moreover, molecular architecture strongly influences water dynamics. MDX, composed of branched amylopectin and helical amylose, undergoes structural rearrangements under small water variations, often transitioning from powder to gel-like states during drying. This explains its higher compression energy compared to the more linear and crystalline BNC fibrils [32]. The asymmetric compression/decompression energies in MDX also reflect the lubricating effect of free water, a mechanism previously observed in dough systems [33].

For BNC powders the energy required for compression was the same as the energy for decompression, while for MDX powders it was observed that the energy required for compression is higher than that for decompression. The energy for decompression is higher for BNC compared to MDX. The decrease in energy for decompression for MDX during the test may be because of the lubricating effect of free water molecules, which also reported in the case of dough and other structures [24]. The rheological behavior can be explained by three main factors: (1) Molecular structure linear cellulose fibrils in BNC vs. branched/amorphous chains in MDX. (2) Water distribution—higher bound water in BNC vs. more free water in MDX. (3) Bioactive incorporation—vitamin B12 reinforced cohesion in MDX but showed negligible effects in BNC.

These findings highlight that rational design of encapsulation carriers should consider not only polymer type and moisture but also the balance of bound vs. free water, since this directly impacts powder compressibility, storage stability, and reconstitution capacity [34].

### 2.3. Cohesion Index (CI)

**Figure 5:**
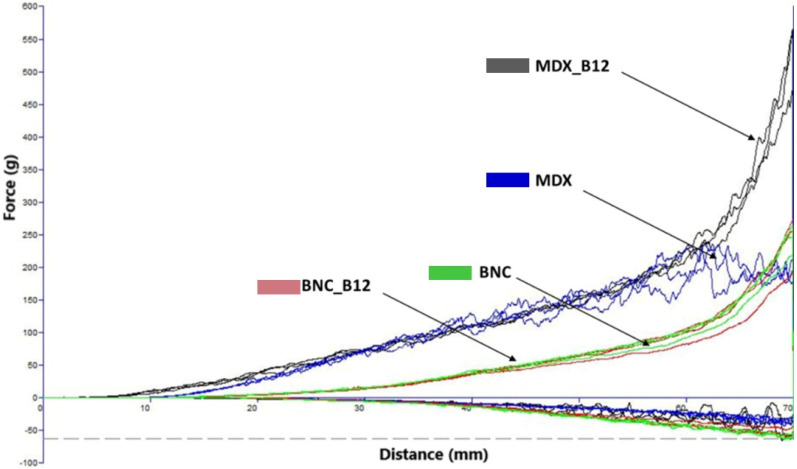
Force–distance profiles from powder analysis (MDX B12: Dark Gray, MDX: Ink Blue, BCN: Green, BNC_B12: Red).

The cohesion coefficient was obtained from Figure 5 and was used to calculate the CI for all powders. The CIs obtained for BNC, BNC_B12, MDX and MDX_B12 were 13.32, 14.27, 7.7, and 13.7, respectively. According to the classification of powders based on the CI [25] the powders were classified as easy flowing for BNC and MDX_B12, free flowing for MDX and cohesive for BNC_B12. These results are consistent with the data obtained for the energy during compression and decompression, supporting the classification from high to low fluidity as follows: MDX > BNC > MDX_B12 > BNC_B12. It is observed that vitamin B12 modifies the CI of the materials which is in agreement with the interactions observed by FTIR. This is evidence that Van der Waals forces are present not only between the encapsulation material and vitamin B12 but also between microparticles of the powder itself.

It is noteworthy that the incorporation of vitamin B12 modified the CI values, which aligns with the FTIR results indicating specific interactions between the vitamin and the encapsulating matrices. These changes suggest that Van der Waals forces occur not only between vitamin B12 and the polymeric carriers but also between microparticles of the powders themselves, influencing the overall cohesion behavior.

In powder technology, the cohesion index is a critical parameter that reflects interparticle interactions, determining both handling and processability. High cohesion is often associated with reduced flowability, which may complicate downstream operations such as mixing, conveying, and tableting [31]. The observed higher CI for BNC_B12 indicates stronger interparticle bonding, potentially due to the hydroxyl-rich surface of cellulose, which can promote hydrogen bonding and enhance solid–solid contact [35]. Conversely, the lower CI of MDX suggests weaker interactions between particles, which is consistent with its branched polysaccharide structure and higher mobility of free water, as reported by Gharsallaoui [36] in the context of encapsulation performance.

Furthermore, the improvement of flowability observed for MDX_B12 compared to pure MDX may be explained by a particle surface modification induced by the vitamin, reducing surface irregularities and enabling more homogeneous packing. Similar phenomena have been described by Turchiuli et al. (2011) [37], who noted that additive incorporation during spray drying can act as a surface-active agent, reducing powder cohesiveness. On the other hand, the increased cohesion in BNC_B12 may compromise its flow behavior, a limitation that should be considered when designing encapsulated systems for large-scale applications. Nonetheless, this strong cohesion could be advantageous in stabilizing the microcapsule structure, thereby protecting sensitive bioactives during storage and handling [38].

According to the Carr Index scale [24], MDX powders are classified as free flowing, BNC powders as easy flowing, MDX_B12 powders as easy flowing, and BNC_B12 powders as cohesive. Thus, the addition of vitamin B12 increased cohesion for both matrices, with the effect being more pronounced for BNC_B12. The higher cohesion of BNC_B12 is related to its crystalline fibrillar nature, which increases the contact area and promotes stronger interparticle interactions compared to the more amorphous MDX particles.

### 2.4. Microstructure

**Figure 6:**
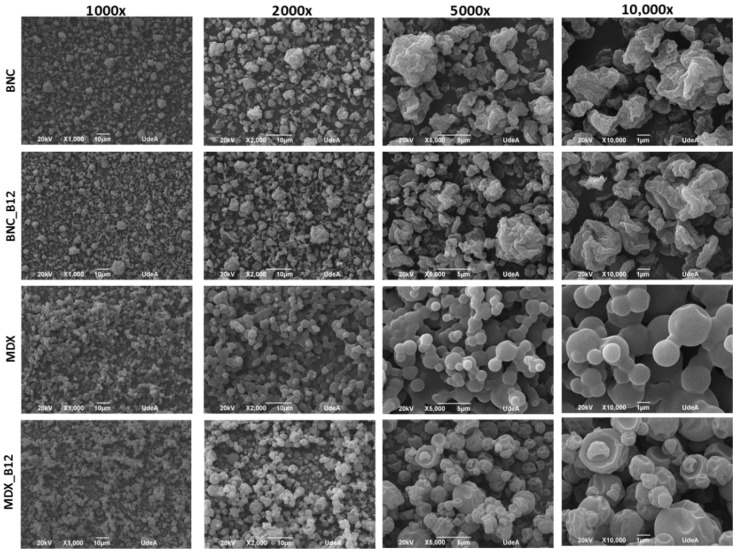
Powder microstructure of BNC, MDX, BNC_B12 and MDX_B12.

Electron microscopy (SEM) has been used to observe the characteristics of agglomerated particles including shape and size. Micrographs of BNC, BNC_B12, MDX and MDX_B12 are shown in Figure 6. Agglomerated particles are visible together with loosely bound and isolated individual particles in the samples. For BNC and BNC_B12 weak bonds were observed, which allow us to measure the particle size. No particle aggregation was observed. SEM micrographs indicate a spherical shape for both, BNC and MDX powders, with particle sizes between ~2 and ~8 µm but most frequent particle sizes between 4 and 5 µm [39]. Large agglomerations are observed in MDX but this agglomeration decrease in MDX_B12, while for BNC and BNC_B12 no agglomerations were observed. The agglomeration behavior is consistent with the results obtained for energy of compression and decompression and maximum force during compression as described above. Vitamin B12 modifies both the BNC and the MDX surface. The roughness of the BNC particles is reduced when B12 is added, which was observed when comparing BNC and BNC_B12 micrographs at 10.000×. On the other hand, MDX particles have a flat surface which is modified when B12 is added, from flat to rough particles.

**Figure 7:**
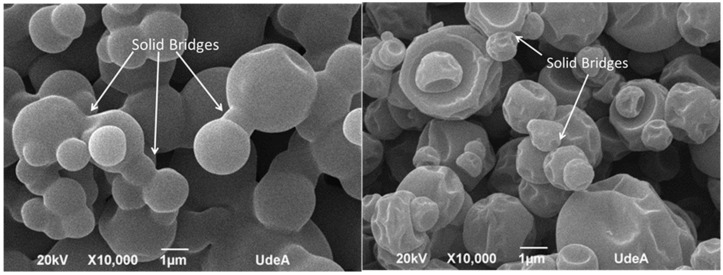
SEM micrography: MDX (left) and MDX_B12 (right).

For MDX solid bridges or “necks” are visible that impair the mechanical strength of the dry granule [20] (Figure 7). It is important to note that this interpretation is a proposed model based on the visual evidence from the micrographs. The structures identified as “bridges” could potentially be distorted particles or artifacts from the sample preparation and drying process. Further analysis would be required to verify the exact nature of these connections and to determine if any vitamin B12 is present within them. The largest solid bridges are present in pure MDX powder but for MDX_B12, the solid bridges of the particles are linked with each other through smaller solid bridges. The MDX solid bridges are large in volume and allow the formation of more voluminous bridges than those of MDX_B12. Solid bridges are less visible in BNC powders.

The observed agglomerates seem to arise from water dissolving and carrying away soluble components from the MDX particles. This particle clumping can actually aid in dissolving or reconstituting the powder in water. The aggregated structure of MDX powders appears to enable the larger clumps to sink and disperse more easily in water as water infiltrates the spaces within the agglomerates, dissolving the connecting “bridges”. This dissolves the connecting “bridges,” which is followed by quick dissolution of the individual particles [21]. Liquid bridges created by water act as a primary binding mechanism in agglomeration. In MDX powders, a thin layer of adsorbed water on the particle surfaces likely smooths out irregularities and reduces the distance between particles. Based on surface texture and particle separation, the analyzed powders rank as follows: BNC = BNC_B12 > MDX_B12 > MDX. During agglomeration, water brings two particles into contact, forming a bond. The agglomerated structure grows as more individual particles attach if water is available. The agglomeration process is dictated by two key factors: the binding mechanism seen in MDX powders, where solid bridges (necks) particles together, and a disruptive force observed in BNC powders that prevents the formation of clumps [20]. Microscopic examination supports the findings from FTIR, cohesion index, and energy of compression/decompression measurements.

The particle size analysis showed that all powders were in the range of 2–10 µm. The mean particle diameters (d50) were 4.5 µm for BNC_B12 and 5.2 µm for MDX_B12. The size distributions were relatively narrow, with d10 values around 2 µm and d90 values around 8–9 µm for all samples. The slightly more uniform and less agglomerated nature of BNC particles likely contributes to their better flow properties observed in the powder flow analysis.

### 2.5. Thermal Stability

**Figure 8:**
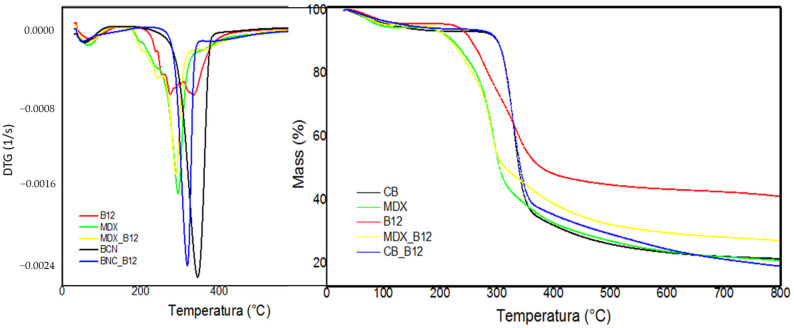
DTG (left) and TGA (right).

The TGA thermal profiles were compared to the corresponding BNC, MDX, B12, BNC-B12 and MDX-B12 (Figure 8). All the samples showed a weight loss between room temperature and 150 °C, corresponding to the loss of powder moisture. A second significant weight loss stage in both TG and DTG curves of each sample is observed between 170 °C and 400 °C. Weight loss for BNC and BNC-B12 begins at 260 °C and ends at 400 °C, which is consistent with Jiang [40]. Weight loss for MDX and MDX_B12 begins at 174 °C and ends at 350 °C which is consistent with Paini [41]. In the pure vitamin B12 the degradation begins at 200 °C, while MDX degradation begins at 174 °C. In the mix of MDX_B12 the degradation also begins at 174 °C, which means that there is no improvement in the thermal behavior of B12. In BNC_B12 it is possible to note how the degradation index follows the degradation index of BNC indicating that both degradations B12 and BNC occur at the same time. Thermal behavior of B12 is improved by BNC, changing the start of degradation temperature of B12 from 200 °C to 260 °C and the maximum temperature degradation from 270 °C to 317 °C.

For BNC_B12, both components degraded within the same temperature range, near the maximum degradation temperature of BNC. In contrast, for MDX_B12, the degradation behavior mirrored that of pure MDX, with no appreciable stabilization effect on vitamin B12. Maximum degradation temperatures were 274 °C for B12, 345 °C for BNC, 317 °C for BNC_B12, 293 °C for MDX, and 288 °C for MDX_B12. These results indicate that cellulose confers a greater stabilizing effect on vitamin B12 than maltodextrin, raising the maximum degradation temperature by 43 °C compared with only 14 °C for MDX.

**Figure 9:**
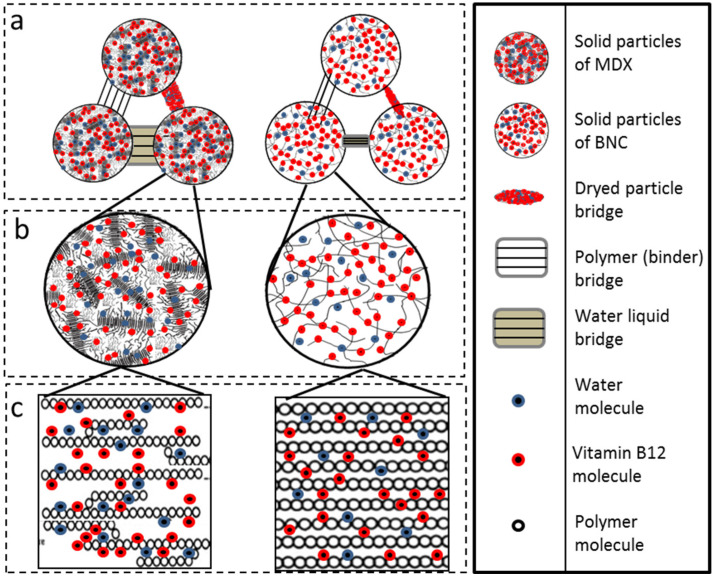
(**a**) Bridges between powder particles, (**b**) web formed between particles, (**c**) absorption of water and B12 particles.

The observed increase in the maximum degradation temperature of vitamin B12 when encapsulated with BNC provides evidence of enhanced thermal stability under extreme conditions. Although such high temperatures are rarely reached during conventional food storage, they are relevant in industrial processes where thermal treatments are applied, such as spray drying, pasteurization, or baking, where short-term exposure to elevated temperatures can significantly reduce vitamin content [42,43]. Therefore, the stabilization effect of BNC suggests that it could contribute to improved retention of B12 during processing steps involving heat. In contrast, under typical storage conditions (ambient or refrigerated temperatures), thermal degradation is less critical, and factors such as light and oxygen exposure become more relevant [44]. Thus, while the improvement observed here is most significant for processing stability rather than long-term storage, it highlights the potential of BNC as an encapsulation matrix in heat-processed food applications (Figure 9).

It should be emphasized that the TGA response of blends is strongly influenced by the predominant mass of the carrier matrix; with B12 present at only ~1 wt%, its individual signal is relatively small. Consequently, the apparent stabilization may partly reflect matrix interactions or overlapping thermal events rather than a direct shift in the intrinsic stability of the vitamin. To confirm these observations, further studies employing model-free kinetic approaches (e.g., Ozawa–Flynn–Wall analysis from multi-rate TGA) or complementary techniques such as DSC are recommended, as these would provide more robust evidence and deeper insights into the degradation mechanisms.

The enhancement of vitamin B12 stability in the presence of BNC can be attributed not only to hydrogen bonding between hydroxyl groups of cellulose and hydroxyl/amine groups of the vitamin, as confirmed by FTIR, but also to structural factors inherent to the carriers. The unbranched, crystalline structure of cellulose limits the retention of free water during spray drying, reducing hydrolytic degradation and contributing to greater thermal protection [45]. In contrast, the branched structure of maltodextrin, composed of amylopectin and amylose, allows higher amounts of water to be entrapped within the particle network [46]. This residual free water, despite high drying temperatures, may facilitate earlier degradation of MDX_B12 systems.

Similar stabilizing effects of cellulose-based carriers on sensitive bioactives have been reported for phenolic compounds and vitamins, where cellulose matrices delayed thermal degradation and improved storage stability [47,48]. The differences observed here between BNC and MDX highlight the importance of carrier selection in encapsulation processes, as the molecular structure and water-binding capacity directly influence thermal protection mechanisms.

Overall, the results confirm that BNC provides superior thermal stabilization to vitamin B12 compared with MDX. This finding is highly relevant for the food and pharmaceutical industries, where the incorporation of thermolabile compounds into cellulose-based matrices could enhance processability and shelf-life without extensive use of synthetic stabilizers [49].

Although the results highlight BNC as a promising carrier for vitamin B12 encapsulation due to its ability to enhance thermal stability, certain practical limitations must be considered before large-scale application. The low aqueous solubility of BNC may restrict its use in some food or pharmaceutical formulations, requiring further chemical or physical modifications to improve dispersibility. Additionally, the production cost and scalability of BNC remain challenges when compared with conventional carriers such as maltodextrin, given the need for controlled fermentation and intensive downstream processing [50,51]. Finally, although this study demonstrated structural protection and improved stability of vitamin B12 within BNC microparticles, the bioavailability of the encapsulated vitamin after ingestion was not assessed and should be addressed in future work. Acknowledging these aspects provides a more balanced perspective on the potential and current limitations of BNC as an encapsulation material.

In this context, the enhanced stability observed is crucial for protecting vitamin B12 during food thermal processing. Future research should also consider short-time, high-temperature simulations (e.g., 200–220 °C for 30–120 s) to better connect TGA results with realistic industrial conditions.

## 3. Materials and Methods

### 3.1. Materials

Bacterial nanocellulose (BNC) was produced in our laboratory using the *Komagataeibacter medellinensis* strain, following previously established fermentation and purification protocols [15]. The final solids content of the BNC suspension was 2.41%, quantified by gravimetry after oven drying at 105 °C. Maltodextrin (MDX) with a dextrose equivalent (DE) of 10 was purchased from Sigma-Aldrich (St. Louis, MO, USA). Vitamin B12 (cyanocobalamin, pharmaceutical grade, lot #B12-54321) was supplied by Merck (Darmstadt, Germany). All other reagents were of analytical grade.

### 3.2. Methods

#### 3.2.1. Preparation of Suspensions for Spray Drying

A BNC suspension (0.5% *w*/*v*) was prepared and mixed with B12. Similarly, a maltodextrin suspension (0.5% *w*/*v*) was prepared and mixed with B12. The ratios were 91 mg of vitamin B12 per gram of BNC or maltodextrin dry matter. Given that the starting BNC material was a 2.41% aqueous dispersion, the concentration of the feed suspension and the relevant ratios were determined based on the dry fiber content within that BNC dispersion. The resulting suspensions underwent mixing using a mechanical agitator at 1800 rpm for a duration of 20 min.

The ratio was selected based on results from previous studies carried out by our group. These preliminary optimization experiments demonstrated that this concentration provided an adequate balance between polymer stability during spray drying, encapsulation efficiency, and vitamin B12 retention [52]. Lower polymer concentrations led to insufficient matrix formation and poor encapsulation yields, while higher concentrations increased viscosity and hindered the atomization process. The chosen ratio thus represents a condition optimized in earlier work to ensure reproducible microparticle formation with adequate physical properties and encapsulation performance.

#### 3.2.2. Spray Drying

Four different powder formulations were prepared as described in Table 2. For the vitamin-loaded samples, vitamin B12 was dissolved in the BNC suspension or MDX solution to a final concentration of 1% (*w*/*w*) based on the total solids content. The mixtures were homogenized using an Ultra-Turrax T-25 (IKA-WERKE Pvt. Ltd., Mumbai, India) at 10,000 rpm for 10 min. The resulting feed solutions were spray-dried using a mini spray dryer B-290 (Büchi Labortechnik AG, Flawil, Switzerland) with a 0.5 mm nozzle. The operating conditions were: inlet temperature of 170 °C, outlet temperature of 90 ± 5 °C, feed flow rate of 5 mL/min, and atomizing gas flow of 473 L/h. The relative humidity (RH) of the drying air was maintained at 30–40%. The powder was collected via a high-performance cyclone, and the collection yield was calculated as the ratio of the mass of powder collected to the mass of solids in the feed solution. The average yield for BNC powders was ~45% and for MDX powders was ~55%. The method was adapted from a previously reported procedure [18].

#### 3.2.3. ATR-FTIR Spectroscopy

Infrared spectra were acquired using an attenuated total reflectance Fourier transform infrared (ATR-FTIR) spectrometer (Waltham, MA, USA), specifically a Nicolet 6700 spectrophotometer fitted with a diamond crystal. Each sample underwent 64 scans at a spectral resolution of 4 cm^−1^, covering a wavenumber range from 4000 to 500 cm^−1^. This analysis was performed on individual samples of pure Vitamin B12, bacterial nanocellulose (BNC), maltodextrin (MDX), as well as the BNC_B12 and MDX_B12 mixtures.

#### 3.2.4. Rheology of the Powders

Flow characteristics of BNC, MDX, BNC_B12, and MDX_B12 powders were evaluated using a TA.XTplus Texture Analyzer (Stable Micro Systems, Godalming, UK) equipped with a Powder Flow Analyser accessory and a 23.5 mm diameter blade probe. This instrument employs rotating blades that move vertically to assess powder behavior. Prior to testing, a consistent volume (140 mL) of each powder was placed into the testing container. The samples underwent three conditioning cycles, involving vertical movement (down and up) of the blades at a speed of 50 mm/s. Compaction behavior was assessed as the rotating blade moved downward, while cohesive properties were measured during the upward blade movement, both at a speed of 50 mm/s. Three compression and cohesion measurements were performed consecutively within each test run, and this entire procedure was repeated three times for each material. The peak force recorded during the downward blade movement represented the maximum force required for compression. Energy expenditure for compression and decompression was calculated based on the positive and negative areas under the force–time curves, respectively, obtained by multiplying the area under the curve by the blade tip speed [8].

#### 3.2.5. Cohesion Index (CI)

The cohesion coefficient (measured in g·mm) represents the energy needed to raise the rotating blade through the powder bed during decompression at a velocity of 50 mm/s. It is quantified by integrating the negative area beneath the force–displacement curve. A standardized rotating blade (48 mm diameter, 10 mm height) was moved vertically, alternating between clockwise and counter-clockwise rotation (TA.XTplus Texture Analyser (Stable Micro Systems, UK) equipped with a Powder Flow Analyser accessory and a 23.5 mm diameter blade probe). Powder flowability was assessed based on the displacement observed during controlled rotation of the blade within the container as described by Mukherjee [53]. The complete methodology is detailed by Abdullah [54]. A low cohesion index (CI) suggests a non-cohesive, free-flowing powder, whereas a high CI indicates a cohesive powder with poor flow characteristics. The CI was calculated using Equation (1):

(1) $$CI\left(mm\right)=\frac{Cohesion\;Coefficient\;(g.mm)}{Sample\;weight\;(g)}$$

#### 3.2.6. Thermal Analysis

Thermal characteristics of spray-dried BNC-B12 and maltodextrin-B12 particles were investigated using a Mettler Toledo TGA/SDTA 851e instrument. Approximately 10 mg of each microparticle type were placed in ceramic crucibles and subjected to a nitrogen atmosphere flowing at 40 mL/min^−1^. Samples were heated from 30 °C to 800 °C at a rate of 10 °C/min. The thermal decomposition temperature was defined as the point at which a significant weight reduction (equal to or exceeding 0.5%) began.

#### 3.2.7. Scanning Electron Microscopy (SEM)

To visualize the shape and particle size distribution of BNC-B12 and maltodextrin-B12, Scanning Electron Microscopy (SEM) was performed using a JEOL JSM 6490 LV instrument (JEOL, Tokyo, Japan) operated at an accelerating voltage of 20 kV. Prior to imaging, samples were sputter-coated with a gold/palladium alloy to enhance conductivity. ImageJ 1.53 e software was employed to determine particle size distribution. At least 150 particles were measured for each sample to calculate the mean particle diameter and the size distribution percentiles (d10, d50, and d90). The choice of SEM for particle size analysis was made to concurrently evaluate morphology and size, which is critical for understanding powder behavior.

#### 3.2.8. Statistical Analysis

The data collected was reported as the average of tests performed in triplicate. ANOVA was used for the analysis of results (SPSS program version 10.0 for Windows). Duncan’s multiple range test was used to test for differences between means.

## 4. Conclusions

This study successfully demonstrated the potential of bacterial nanocellulose (BNC) as an effective encapsulating agent for vitamin B12. The powders produced with BNC exhibited superior flow properties and comparable thermal stability to those made with the conventional encapsulant, maltodextrin. The micrometric, spherical particles obtained confirm the suitability of the spray-drying process. Key findings supported by our data include the confirmation of vitamin–matrix interactions via ATR-FTIR, significantly better flowability of BNC powders as shown by lower Cohesion Index values, and an increase in the vitamin’s thermal degradation temperature by up to 30 °C when encapsulated in BNC.

However, this study has limitations. Encapsulation efficiency, loading capacity, and reconstitution properties were not quantitatively determined. Future research should focus on these aspects, as well as on performing density and color analyses. Additionally, conducting in vitro release and bioaccessibility studies would provide a more complete understanding of BNC’s performance as a carrier for dietary supplements. Further investigation using techniques like model-free kinetics (from multi-rate TGA) and DSC would help corroborate the thermal stability findings. The conclusions on thermal stability are based on TGA and could be strengthened by complementary techniques. Furthermore, the industrial readiness of these powders cannot be fully assessed without these data and studies on storage stability.

In conclusion, BNC stands as a viable and promising biopolymer for the food and pharmaceutical industries, particularly for encapsulating sensitive bioactive compounds.

## Figures and Tables

**Table 1:** Main ATR-FTIR band assignments for the pure components.

BNC		
Wavenumber cm^−1^	Vibration	Reference
3339	OH	[7,8,10]
1431	CH2 sym	[11,12]
1425	OH def vibr	[13]
1236	O–H in-plane	[12,14]
1034	C-H wag vibr	[15]
644	OH def in plan	[16]
MDX		
Wavenumber cm^−1^	Vibration	Reference
3284	O-H	[17]
2187–2018	CO	[18]
1362	COC	[19]
853	CH2	[20]
B12		
Wavenumber cm^−1^	Vibration	Reference
2991–2932–2877–2825	C-H str	[17,21]
2138	C Ξ N	[9]
1658	CO asim	[22]
1493–810–667	C = C	[23]
1544	C = N	[7,13]
848–707–578	pyranoid ring	[24,25]

**Table 2:** Composition of the powder formulation prepared by spray drying.

Sample ID	Encapsulant Matrix	Active Compound	Vitamin B12 Concentration (% *w*/*w* of Total Solids)
BNC	Bacterial Nanocellulose	None	0
BNC_B12	Bacterial Nanocellulose	Vitamin B12	9
MDX	Maltodextrin	None	0
MDX_B12	Maltodextrin	Vitamin B12	9

## Data Availability

The original contributions presented in this study are included in the article. Further inquiries can be directed to the corresponding author.

## Abbreviation

BNC	Bacterial nanocellulose
MDX	Maltodextrin
B12	Vitamin B12 (cyanocobalamin)
ATR-FTIR	Attenuated Total Reflectance Fourier Transform Infrared Spectroscopy
SEM	Scanning Electron Microscopy
TGA	Thermogravimetric Analysis
DTG	Differential Thermogravimetric Analysis
CI	Cohesion Index
DE	Dextrose Equivalent
RH	Relative Humidity
